# Evaluating the Effects of Metallic Waste on the Structural and Gamma-Ray Shielding Properties of Epoxy Composites

**DOI:** 10.3390/polym16101415

**Published:** 2024-05-16

**Authors:** Sitah Alanazi, Mohammad Hanfi, Mohammad W. Marashdeh, Mamduh J. Aljaafreh, Karem A. Mahmoud

**Affiliations:** 1 Department of Physics, College of Sciences, Imam Mohammad Ibn Saud Islamic University (IMSIU), P.O. Box 90950, Riyadh 11623, Saudi Arabia; sfenazi@imamu.edu.sa (S.A.);; 2 Department of Life Safety, Institute of Fundamental Education, Ural Federal University, Ekaterinburg 620002, Russia; 3 Nuclear Materials Authority, P.O. Box 530, El-Maadi, Cairo 11728, Egypt; 4 Department of Nuclear Power Plants and Renewable Energy Sources, Ural Power Engineering Institute, Ural Federal University, Ekaterinburg 620002, Russia

**Keywords:** metallic waste, radiation parameters, epoxy resin, (MCNP) simulation

## Abstract

The objective of the research is to develop novel materials that are both inexpensive and have a low density, while also being able to endure the transportation of γ-photons with low-to-medium energy levels. The outcome consisted of four epoxy resins that were strengthened with different quantities of heavy metallic waste. The density of the formed composites improved from 1.134 ± 0.022 g/cm^3^ to 1.560 ± 0.0312 g/cm^3^ when the waste content was raised from 0 to 40 weight percent. The theoretical investigation was determined using Monte Carlo (MCNP) simulation software, and the results of linear attenuation coefficient were justified experimentally in a low and medium energy range of 15–662 keV. The mass attenuation coefficient results in a low gamma energy range (15–122 keV) varied in between 3.175 and 0.159 cm^2^/g (for E-MW0 composite) and in between 8.212 and 0.164 cm^2^/g (for E-MW40 composite). The decrease in mass attenuation coefficient was detected in a medium gamma photon energy range (122–662 keV) with 0.123–0.082 cm^2^/g (for E-MW0 composite) and 0.121–0.080 cm^2^/g (for E-MW40 composite). The density of the enhanced composites influenced these parameters. As the metallic waste composition increased, the fabricated composites’ half-value thickness decreased. At 15 keV, the half-value thickness decreased from 0.19 to 0.05 cm. At 59 keV, it fell from 2.70 to 1.41 cm. At 122 keV, it fell from 3.90 to 2.72 cm. At 662 keV, it fell from 7.45 to 5.56 cm. This decrease occurred as the heavy metal waste concentration increased from 0 to 40 wt.%. The study indicates that as metallic waste concentrations rise, there is a rise in the effective atomic number and a decline in the buildup factors.

## 1. Introduction

Radiation is divided into two types: ionizing and non-ionizing. Radiation is a kind of energy that originates from a source and moves across space, with the ability to pass through different substances. Various forms of radiation possess varying capacities to ionize substances [1,2,3]. Non-ionizing radiation, including visible light, radio waves, and microwaves, lacks the energy required to displace electrons from an atom [4,5]. Ionizing radiation, a form of electromagnetic radiation, has higher energy levels than non-ionizing radiation. This higher energy allows it to remove electrons from atoms, resulting in the formation of ions. Ionizing radiation is composed of several particles and photons, such as X-rays, gamma rays, alpha rays, and beta rays [6,7]. A growing number of industries, including nuclear facilities, medical diagnostics, research labs, processing of foods, waste storage, biological studies, defect detection in manufacturing, medical imaging and treatment, space exploration, and high-energy physics experiments, are using high-energy ionizing radiation, especially gamma rays [8,9]. Because gamma rays are powerful and penetrating, accidental or inadvertent exposure to them may be damaging to people, the environment, and items. Such exposure may result in radiation illness, organ damage, cancer, cell mutations, component failure, and other negative impacts on human health and general well-being [10,11]. As a result, it is critical to shelter the environment and people from radiation’s damaging effects using the following four principles: activity, time, distance, and shielding. Extending the distance and shortening the exposure time can help reduce the radiation dose from the source.

Despite having inadequate mechanical qualities overall, polymers are utilized in situations where flexibility is necessary. But when they are stressed or loaded heavily, they often distort. Reinforcement techniques may be used to add inorganic particles to increase mechanical qualities, including modulus, stiffness, and tensile strength. These qualities may be tailored by changing the filler particles’ volume percent, shape, and size. Mechanical characteristics may be significantly improved by reinforcing a polymer matrix with nanofillers that have a higher aspect ratio and stiffness [12,13]. Recently, many researchers have sought to develop novel methods for recycling and waste management using polymer composites [14]. The recycling process involves incorporating various filler components into recycled polymers that are biodegradable, with the goal of creating environmentally friendly and sustainable products [15,16]. Several efforts have been made to incorporate biodegradable fillers that can improve composite performance [17]. Using reusable fibers to create thermoplastic composites, other researchers tried to decrease the requirement for additives while keeping a sustainable and unpolluted environment [18,19,20]. The goal of this study is to develop novel, lower-density composite materials that can block low- and intermediate-energy gamma rays. These materials might be used in the medical field, among other sectors. As a result, polymer composites were fabricated, and the materials’ shielding performance was assessed using the narrow beam transmission technique.

## 2. Materials and Methods

### 2.1. Fabricating the Resin with Heavy Metallic Waste

Industries have challenges in managing the substantial metallic waste generated by the heavy equipment industry due to the need for recycling to ensure proper disposal. This research utilizes heavy metallic wastes with chemical compositions specified in Table 1 to determine how to enhance the linear attenuation coefficient of gamma radiation in low-cost epoxy resin. The objective was to develop new composites that are effective in shielding against low to intermediate gamma energy. As a result, using a vertical mixer, various concentrations of heavy metallic debris were combined with the epoxy resin for 20 min. SlabDOC in Ivanovo, Russia, provided the high-quality (98%) epoxy resin and hardener utilized in the investigation. The molecular weights of the hardener and epoxy resin were 23.38 and 392 g/mol, respectively. The composites were made using a weight/weight process, with exact measurements of the hardener, epoxy resin, and heavy metal wastes made using an electronic balance that could quantify changes in concentration as little as 0.01 mg. The heavy metal wastes (designated as E-MW0%, E-MW10%, E-MW20%, and E-MW40%) were added to the composites at weight concentrations of 0%, 10%, 20%, and 40%, respectively. In every sample, the hardener-to-epoxy resin ratio was maintained at 0.5. Poured into a cylindrical silicon mold with a set 3 cm diameter, the mixture was left to harden overnight at +25 °C. A density meter from Dongguan, China, the MXBAOHENG MH 300A, was used to measure the densities of the synthetic composites. The measured uncertainty with the MH300 was determined to be 0.001 g/cm^3^. Based on the Archimedean principle, the MH300 density meter computes the composite density using Equation (1), where *W_a_* and *W_L_* stand for the composite weights in dry air and submerged liquid, respectively, as well as the density of water: *ρ_L_* = 1 g/cm^3^ [21].

(1) $$Density\;\left(\rho ,\;\frac{g}{{cm}^{3}}\right)=\frac{\left(W_{a}-W_{L}\right)}{W_{a}}\;\rho_{L}$$

The Tescan MIRA3 scanning electron microscope (SEM) manufactured in Brno, Czech Republic, was used to determine the spatial arrangement of metallic debris inside the epoxy composites. The scanning electron microscope (SEM) captured images at a magnification of 553x using a 20 kV acceleration voltage. The chemical composition of the epoxy composites, including the generated metallic waste, was analyzed by energy dispersive X-ray (EDX) analysis, as described in Table 1.

### 2.2. Gamma-Ray Shielding Capacity Examination

**Figure 1:**
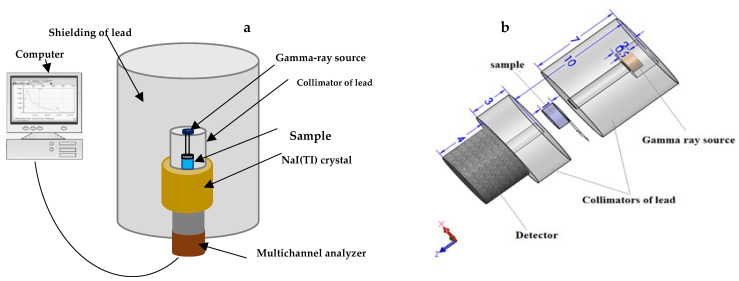
(**a**) Diagram illustrating the procedure of measuring the linear attenuation coefficient using the narrow beam transmission technique and (**b**) a three-dimensional representation of the MCNP-5 input file.

Monte Carlo simulation (MCNP-5) software was used to validate the radiation shielding parameters that were acquired from the experiment [22]. The gamma-ray energies employed in the MCNP simulation were selected to correspond with the actual energies used in the experimental experiments (Figure 1a), such as 59, 80, and 662 keV. The MCNP code was linked to the ENDF/B-V.8 nuclear database, which has the necessary interaction cross-sections to evaluate the radiation shielding abilities of epoxy composites doped with fabricated metallic waste. An input file describing the properties of each simulation component—such as the cell, surface, material, significance, source, and cutoff maps—must be produced prior to running these kinds of simulations. Figure 1b displays the 3D representation of this input file. According to the data in the input file, a lead cylinder served as a barrier between the radioactive source, detector, collimator, and samples throughout the simulation. The dimensions of this cylinder were 5 cm width, 20 cm diameter, and 30 cm height. Next, a radioactive source with dimensions of 2 cm in diameter and 0.5 cm in thickness was positioned in the middle of the outer lead cylinder (at coordinates POS = 0 0 0). Gamma photons (parameter PAR = 2) along the positive Z-axis (coordinates AXS = 0 0 1) might be emitted by the source. The source card contained information about the radioactive source’s distribution and emission probability as well as the type of radiation and the source’s location. Using a lead collimator, the radioactive source’s photon flux was focused onto the epoxy samples doped with metal waste. The earliest lead collimator described was 5 cm in diameter and 7 cm high, with a 1 cm narrow vertical slit. The material card (see Table 1) included information on the density and chemical composition of the tested epoxy samples that contained metallic debris. The fabricated samples had a cylindrical form with a diameter of 3 cm and a height of 1 cm, in accordance with the surface map data. After interacting with the electrons and atoms in the composites, the photon flux dispersed and underwent collimation by a different collimator before entering the detector. This second collimator’s dimensions were 3 cm in height and 5 cm in diameter. It was also cylindrical. By measuring the average flux per cell of the fabricated composites, the average track length (ATL) of gamma rays inside the composites was calculated. In this work, we used Tally F4 to calculate the necessary ATL of gamma photons in Monte Carlo (MCNP) simulation software to validate the radiation shielding parameters acquired from the composites created in this experiment [22]. It was configured to handle 108 histories of photon–electron interactions. The synthesized composites automatically created an output file that recorded the simulated ATL of gamma rays. The output file displays a relative inaccuracy within a range of approximately 1% [23,24]. The simulated ATL was then used to calculate the values of µ and µm.

The mass attenuation coefficient (µm, cm^2^/g) for the produced composites was then calculated by using the observed µ values as per Equation (2). A gamma-ray spectrometer with a NaI (Tl) crystal was used to obtain and figure out the linear attenuation coefficient (µ, cm^−1^; Equation (3)) for different gamma-ray energies given off by radioactive sources like Cs-137, Am-241, and Ba-133 [25,26].

(2) $$µ\;\left(cm^{-1}\right)=\frac{1}{x}\ln\left(\frac{I_{o}}{I_{t}}\right)\;$$

(3) $$µ\;\left(cm^{-1}\right)=\frac{1}{x}\ln\left(\frac{I_{o}}{I_{t}}\right)\;$$

The half-value thickness (Δ0.5, cm) indicates the depth of the composite material that reduces the photon flux of the radioactive source by 50%. It is inversely proportional to the µ values, with denser composites having higher µ values than less dense composites [27,28].

(4) $${∆}_{0.5}\left(cm\right)=\frac{ln(2)}{µ\left(cm^{-1}\right)}$$

The radiation protection efficiency (RPE, %) measures the absorbed activity of the radioactive source within the created composites (*Ia*) in relation to the original radioactive source activity (*Io*) and can be determined using Equation (5).

(5) $$RPE(\%)=\frac{I_{a}}{I_{o}}\times 100$$

The study examined the effective atomic number (Z_eff_), comparable atomic numbers (Z_eq_), and factors related to buildup (such as buildup factor (EBF) and energy absorption buildup factor (EABF)) using the Phy-X/PSD program [29].

## 3. Results and Discussion

**Figure 2:**
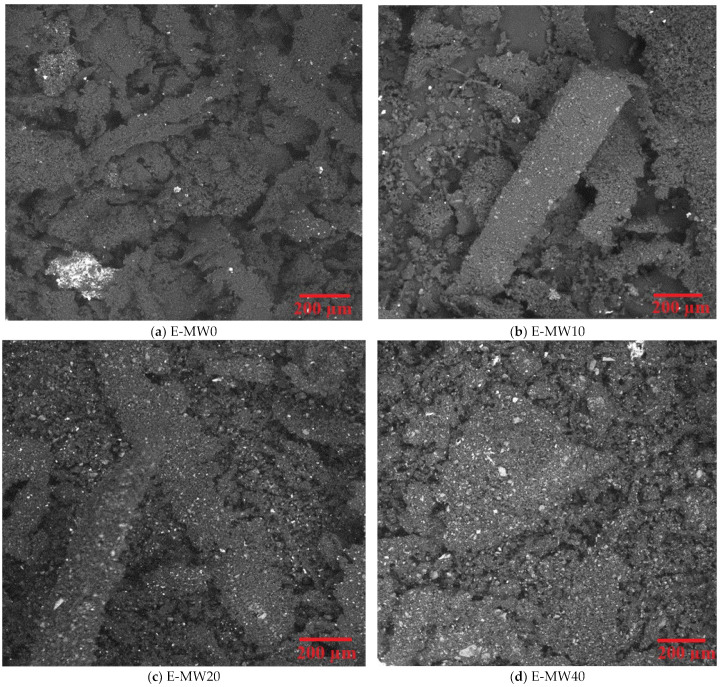
SEM images for the E-MW0, E-MW10, E-MW20, and E-MW40 fabricated samples.

In the context of the aforementioned study, the use of a scanning electron microscope (SEM) provided valuable insights into the microstructure of the polymer doped with heavy metal waste materials. SEM is a powerful tool for high-resolution imaging and analysis of the surface morphology of materials. Using SEM, the distribution of heavy metallic debris particles within the epoxy resin matrix was observed. This allowed a detailed examination of the interface between the polymer matrix and the metallic debris, providing information on the dispersion and clustering of the waste materials. Figure 2 displays scanning electron microscopy (SEM) images that demonstrate a consistent distribution of metallic waste fillers throughout the epoxy-based composites. Figure 2a depicts the epoxy composites in their pure form, without any inclusion of metallic waste fillers. Figure 2b,c demonstrate a significant rise in the scattered fillers when the concentrations of metallic waste are increased, with the metallic waste fillers being distributed in the following order: E-MW40 > E-MW20 > E-MW10 > E-MW0.

The experimental measurements showed that an increase in the concentration of metallic waste within the fabricated composites results in a corresponding increase in density. Table 1 shows that an increase in the concentration of metallic waste between 0 and 40% by weight results in a density of 1.56 ± 0.0374 g/cm^3^ for the developed composite, which is an increase compared to 1.134 ± 0.0238 g/cm^3^. As shown in Table 1, the high concentrations of Ca, Fe, and Si in the metallic waste used to improve the epoxy resin are the reason for the increase in composite density.

**Figure 3:**
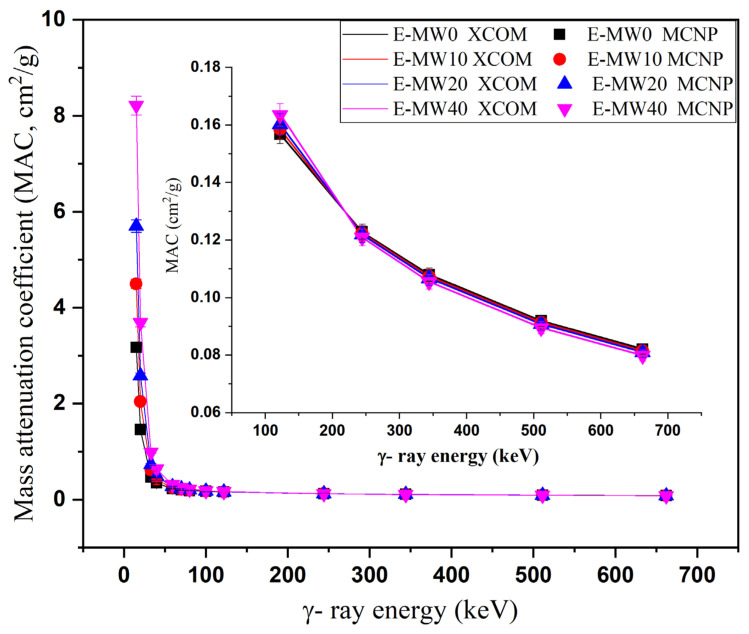
Mass attenuation coefficient variation of the fabricated samples vs. incident gamma energy of photons at photon energy.

Figure 3 shows the effects of γ-photon energy on the simulated MAC for the fabricated E-MW samples over a photon energy interval ranging from 15 to 662 keV. In the aforementioned energy interval, the γ-photons interact with the composite electrons with two different interaction modes (i.e., photoelectric interaction (PE) and Compton scattering (CS)). Over the low energy interval from 15 to 122 keV, the PE interaction is the leading one. Since the cross-section of the interaction within the PE interval varies with E^−3.5^, a large reduction in MAC was observed for all fabricated composites in Figure 3. Increasing the photon energy from 15 to 122 keV causes a reduction in MAC between 3.175–0.159 cm^2^/g (for E-MW0 composite), 4.496–0.159 cm^2^/g (for E-MW10 composite), 5.702–0.160 cm^2^/g (for E-MW20 composite), and 8.212–0.164 cm^2^/g (for E-MW40 composite). The inset of Figure 3 shows the variation of MAC versus γ-ray energy over the CS interval, which extends from 244 to 662 keV. Due to the proportionality of the CS cross-section with E^−1^, an exponential decrease in MAC was observed. The MAC decreased between 0.123–0.082 cm^2^/g (for E-MW0 composite), 0.122–0.082 cm2/g (for E-MW10 composite), 0.122–0.081 cm^2^/g (for E-MW20 composite), and 0.121–0.080 cm^2^/g (for E-MW40 composite) as the photon energy increased between 244 and 662 MeV, respectively. Polymeric materials are unsuitable for shielding the high-energy γ-photons, which is the main reason for selecting the energy interval between 15 and 662 keV. Figure 3 also shows an agreement between the simulated data with the MCNP-5 code and those calculated with the Phy-X/PSD program. The difference between the two results was within ±2%. The simulated LAC for the fabricated samples was then compared with the experimentally measured LAC for the fabricated composites at γ-ray energies of 59.5 keV, 80 keV, and 662 keV, as shown in Table 2. The difference between the simulated and experimental results is high for the Ba-133 radioactive source with an energy of 80 keV, where the difference varied between 8–15%. This high difference can be attributed to the low activity of the source used and to errors in the chemical composition of the fabricated composites. Then, for the radioactive sources Am-241 (59 keV) and Cs-137 (662 keV), the difference between experimental and simulated data was in the range of ±10%.

**Figure 4:**
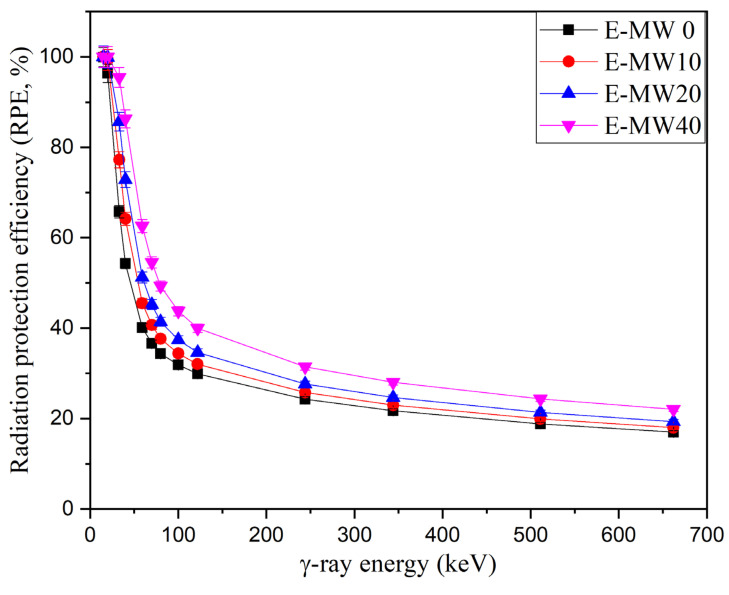
Variation of dependence of the radiation protections efficiency (RPE, %) on the γ-photon energy.

The RPE of the fabricated epoxy-based composites was evaluated, and its dependence on γ-photon energy is clarified in Figure 4. Figure 4 shows how the RPE behaves as the energy of the γ-photon increases between 15 keV and 662 keV. The RPE values are related to the number of photons absorbed within the fabricated composite during the interaction, and Figure 4 shows that the RPE for 2 cm thick fabricated composites is higher than 95% within the energy interval between 15 keV and 20 keV for all fabricated composites. Then, the above values decrease with increasing photon energy until they reach 16.99%, 18.00%, 19.32%, and 22.05% for samples E-MW0, E-MW10, E-MW20, and E-MW40, respectively. This behavior is related to the penetration power and interaction cross-section of γ-photons, where with the increase of photon energy, the penetration power of γ-photons increases, and both the interaction cross-section and the interaction probability for the traveling γ-photons decrease. As a result, the number of interactions decreased, followed by a decrease in the number of photons absorbed within the composite and an increase in the number of transmitted photons. The net result is a reduction in RPE for the fabricated composites as the photon energy increases. 

**Figure 5:**
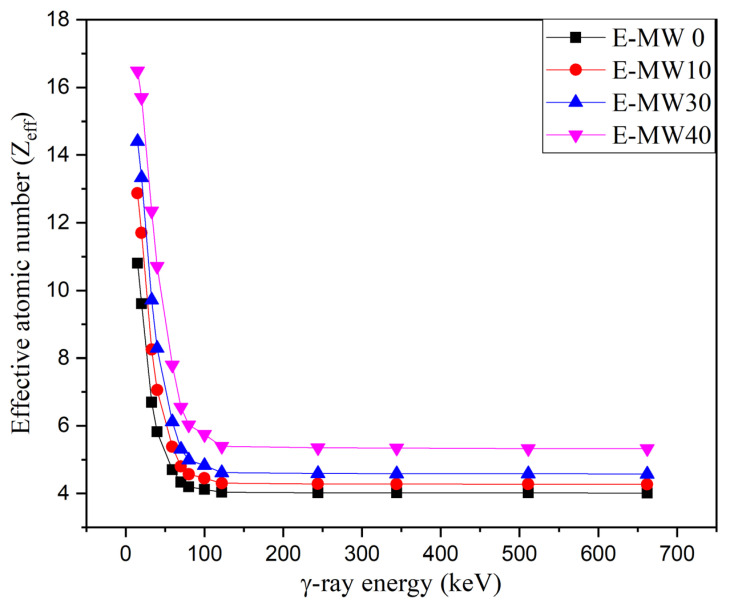
Relationship between the effective atomic number (Z_eff_) and the energy of γ-photon.

The effective atomic number (Z_eff_) for the fabricated composites is calculated using the Phy-X/PSD program to know which element has the same shielding capacity as the fabricated composites at different energies. Figure 5 shows the variation of Z_eff_ versus γ-photon energy, where the highest Z_eff_ values were observed at low photon energies (i.e., 15 keV). According to the data presented in Figure 5, the Z_eff_ values are ≈ 11, 13, 14, and 16 for the prepared composites E-MW0, E-MW10, E-MW20, and E-MW40, respectively. This means that the above-mentioned composites E-MW0, E-MW10, E-MW20, and E-MW40 have shielding effectiveness similar to the elements Na, Al, Si, and S at γ-photon energy of 15 keV. The increase in photon energy then causes an exponential decrease in Z_eff_ values, with Z_eff_ dropping to 4 (for E-MW0 and E-MW10 composites) and 5 (for E-MW20 and E-MW40 composites) at 662 keV.

**Figure 6:**
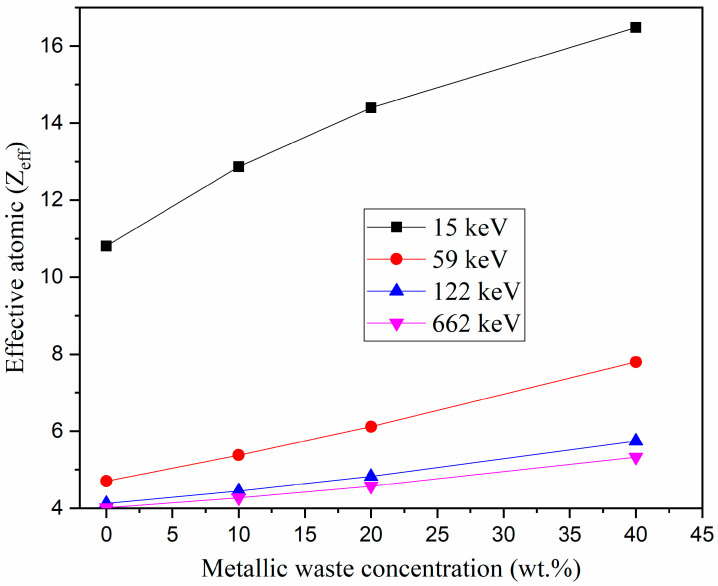
Dependence of effective atomic number (Z_eff_) on the metallic waste concentration.

In addition, the chemical composition (i.e., the concentration of metallic waste) affects the values of Z_eff_, as shown in Figure 6. The addition of metallic wastes to the epoxy resin increases the density of the synthesized composites because the epoxy resin is mainly composed of C and H, but the metallic wastes used in this work are rich in Fe, Si, Ca, and Al. The addition of metallic wastes to the epoxy resin also increases the electron density and Z_eff_ of the prepared composites, as shown in Figure 6. The addition of metallic wastes at concentrations of 0 and 40 wt.% increases the Z_eff_ values to between 10.8–16.5 (at 15 keV), 4.7–7.8 (at 59 keV), 4.1–5.7 (at 122 keV), and 4.0–5.3 (at 662 keV).

**Figure 7:**
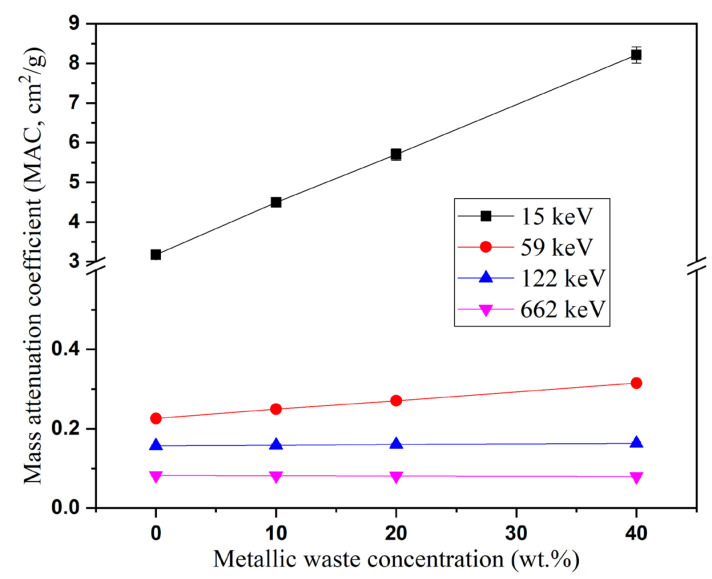
Reliance impact of weight percentage concentrations of metallic waste on the mass attenuation coefficient at a given γ-photon energy.

The dispersion of metallic waste in the epoxy structure also affected the radiation shielding properties. Figure 7 shows that the metallic waste concentration increased the MAC values for the fabricated composites. Since the interaction cross-section for γ-photons varies with $Z_{eff}^{4-5}$ and Z_eff_ in the PE and CS intervals, the MAC values are greatly increased in the PE interval as the metallic waste concentration increases. According to the data in Figure 7, increasing the metallic waste concentration between 0 and 40 wt% causes an increase in the MAC of the fabricated composites between 3.175 and 8.212 cm^2^/g (at 15 keV), 0.226–0.315 cm^2^/g (at 59 keV), and 0.157–0.164 cm^2^/g (at a122 keV). In contrast, there is no significant change in the MAC at the CS interval, where the MAC varied between 0.082–0.080 cm^2^/g (at 662 MeV) as the metallic waste increased from 0 to 40 wt%. The epoxy resin and its modified composites have shielding properties unsuitable for the middle range of high gamma-ray energies. These composites can only be used for low gamma-ray shielding applications. The LAC has the same behavior as the MAC at all energies studied. The addition of metallic wastes between 0 and 40 wt% increases the LAC between 3.600–12.811 cm^−1^ (at 15 keV), 0.256–0.491 cm^−1^ (at 59 keV), 0.178–0.255 cm^−1^ (at 122 keV), and 0.093–0.125 cm^−1^ (at 662 keV), respectively.

In order to determine how well metallic waste-doped epoxy resin composites shield radiation, we compared the µm values of the E-MW composites with those of other polymers and commercial materials from previous studies [30,31,32,33,34,35,36,37,38,39], as shown in Table 3. The µm values for the E-MW composites at 662 keV were 0.082 cm^2^/g, 0.082 cm^2^/g, 0.081 cm^2^/g, and 0.080 cm^2^/g for the E-MW0, E-MW10, E-MW20, and E-MW40 fabricated composites, respectively. According to the results in Table 3, HDP nano-PbO (50%) had the highest µm value of 0.1140 cm^2^/g among the composites compared at a γ-photon energy of 662 keV.

**Figure 8:**
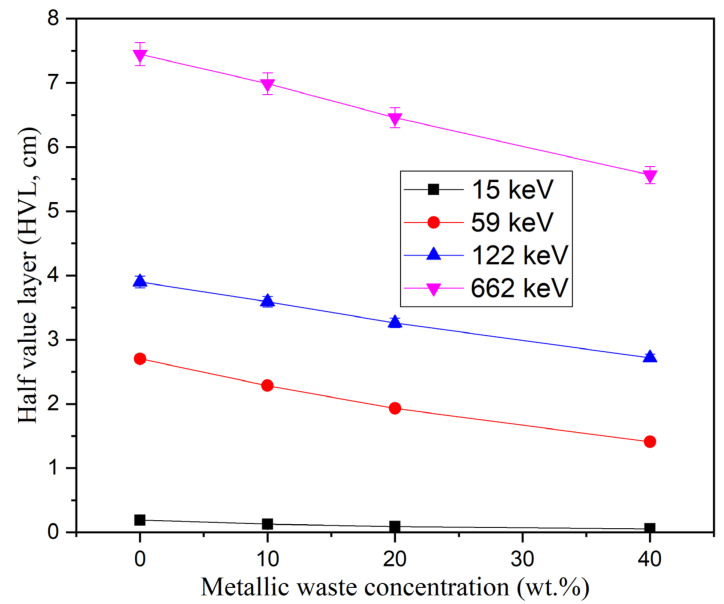
Dependence influence of the heavy metallic waste concentrations on half-value thickness (Δ0.5, cm) at some fixed γ-photon energy.

**Figure 9:**
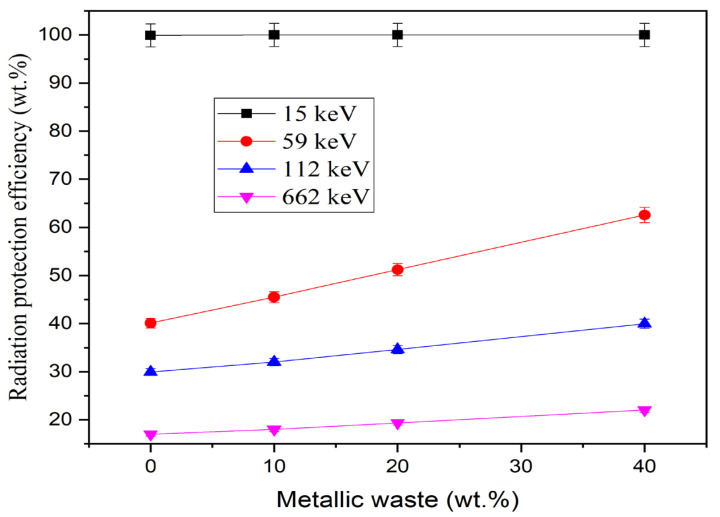
The fabricated composites’ radiation protection effectiveness (RPE,%) as a function of metallic waste.

The HVL is inversely proportional to the LAC, which is the main reason for the decrease in the HVL with increasing metallic waste concentration, as shown in Figure 8. Increasing the metallic waste concentration between 0 and 40 wt% decreases the HVL for the fabricated composites between 0.19–0.05 cm (at 15 keV), 2.70–1.41 cm (at 59 keV), 3.90–2.72 cm (at 122 keV), and 7.45–5.56 cm (at 662 keV). In addition, the RPE of the fabricated composites was affected by the addition of metallic waste concentrations to the epoxy resin, with the RPE of the fabricated composites being enhanced by increasing the metallic waste concentrations. Figure 9 shows an increase in the RPE of 2 cm thick fabricated composites between 40.1–62.6% (at 59 keV), 29.9–40.0% (at 122 keV), and 17.0–22.1% (at 662 keV). In addition, at 15 keV, the RPE is nearly 100% for all composites due to the PE interaction, which consumes the energy of the photons during the interaction.

**Figure 10:**
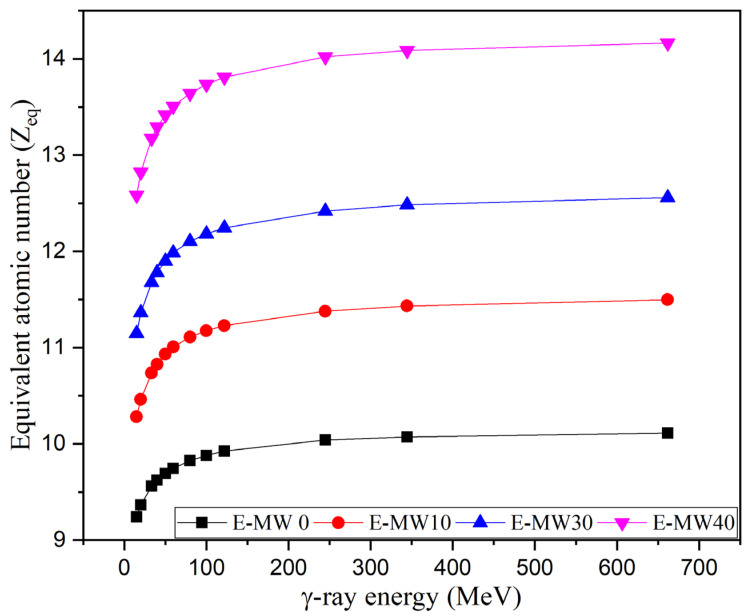
The relationship between the γ-photon energy and the equivalent atomic number (Z_eq_).

To calculate buildup factors (BUFs: EBF and EABF), it is necessary to calculate the equivalent atomic number (Z_eq_), which describes the multielement composite in terms of its equivalent element. The Z_eq_ values are proven by dividing the MAC in CS interval by the total MAC of the material. Figure 10 shows that the increase in γ-photon energy between 15 keV and 662 keV causes an increase in the Z_eq_ for the fabricated composites. The Z_eq_ starts very low at 15 keV, where the Z_eq_ takes small values due to the predominance of PE and the CS of 9.24, 10.28, 11.15, and 12.58 for samples E-MW0, E-MW10, E-MW20, and E-MW40, respectively. After that, the increase in γ-photon energy increases the CS interaction, and the MAC in CS increased compared to the total MAC for each composite. Therefore, the Z_eq_ values increased until the γ-photon energy reached 100 keV. The increase in photon energy in the interval between 100 and 662 keV causes a slight increase in the Z_eq_ values by 2.9%, 3.4%, 3.6%, and 3.7%, respectively, for samples E-MW0, E-MW10, E-MW20, and E-MW40. Based on the Z_eq_ values, the BUPs values for the fabricated composites were estimated.

**Figure 11:**
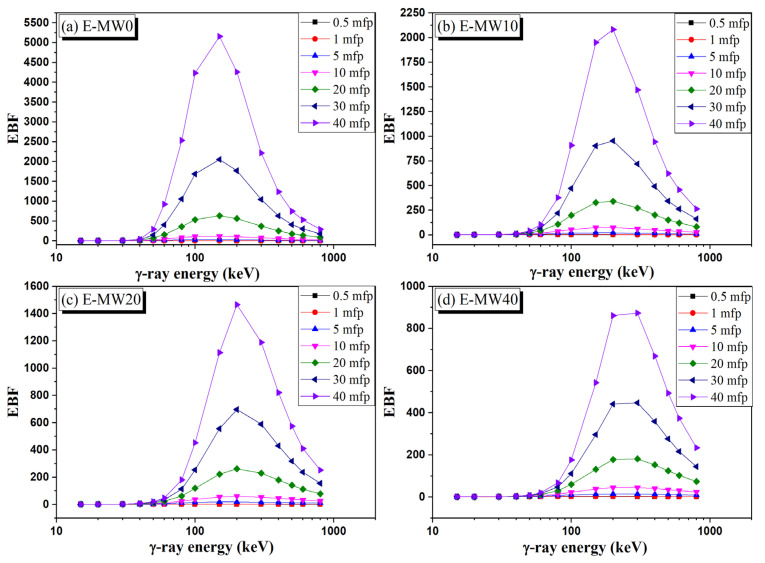
Dependence of exposure buildup factor (EBF) on the γ-photon energy.

**Figure 12:**
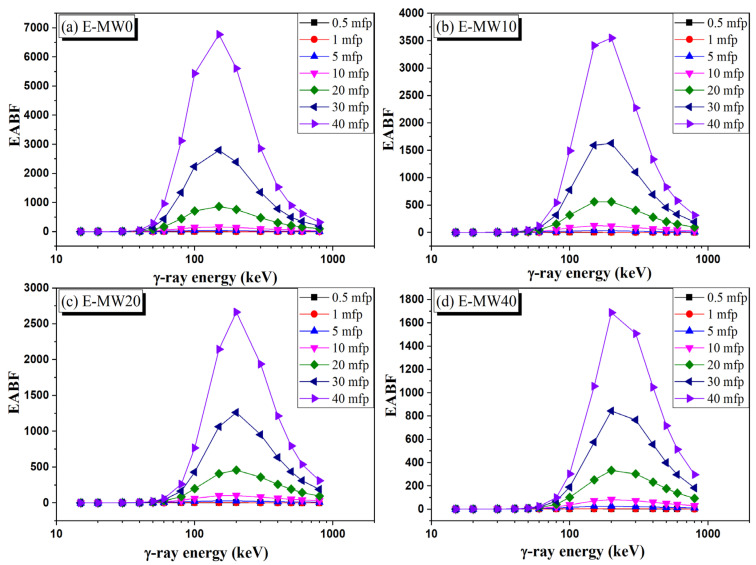
Dependence of absorption buildup factor (EABF) on the γ-photon energy.

The variation of EBF and EABF versus the γ-photon energy over the energy interval between 15 and 662 keV is illustrated in Figure 11 and Figure 12. Both EBF and EABF are very low at gamma photon energy lower than 80 keV due to the disappearance of CS interaction. Then, raising the photon energy above 100 keV, the EBD and EABF values were increased highly due to the increase in CS interaction. Near 150 keV, the EBF and EABF reached maximum values and then began to decrease again at photon energy greater than 200 keV. The occurrence of the CS interaction at lower energy is attributed to the low Z_eff_ of the fabricated composites. Figure 11 and Figure 12 show that the increase in metallic waste concentrations decreases the BUFs (EBF and EABF values) recorded for the fabricated samples, where the BUFs for E-MW0 > E-MW10 > E-MW20 > E-MW40. Also, the increase in the source-detector distance increases the BUF, where the BUFs at a source-detector distance follow the pattern of 40 mfp > 20 mfp > 10 mfp > 5 mfp> 0.5 mfp.

## 4. Conclusions

This study investigated how heavy metallic wastes affect the γ-ray shielding properties of epoxy resin. The density of the composite rose from 1.134 ± 0.0238 g/cm^3^ to 1.56 ± 0.0374 g/cm^3^ when metallic waste was up to 40 wt.%. The SEM analysis shows a homogenous distribution for the metallic waste within the epoxy-based composite. The influence of metallic waste concentrations on γ-ray shielding properties of fabricated epoxy-based composites was evaluated using Monte Carlo simulation and experimental measurements. Both methods affirmed the enhancement of fabricated composites’ mass and linear attenuation coefficients by raising the metallic waste concentration. The mass attenuation coefficient increased between 3.175–8.212 cm^2^/g (at 15 keV), 0.226–0.315 cm^2^/g (at 59 keV), and 0.157–0.164 cm^2^/g (at 122 keV) when the metallic waste concentration increased between 9 and 40 wt.%, respectively. Additionally, the radiation protection efficiency for the fabricated composites was enhanced between 40.1–62.6% (at 59 keV) and 29.9–40.0% (at 122 keV) by raising the metallic waste concentration to 40 wt.%. The study shows an increase in the effective atomic number and a decrease in the buildup factors by increasing the metallic waste concentrations.

## Figures and Tables

**Table 1:** The fabricated waste-based epoxy samples’ density and composition of chemicals.

	Elemental Abundance			
	E-MW0	E-MW10	E-MW20	E-MW40
H	0.0697	0.0627	0.0557	0.0418
C	0.4401	0.3961	0.3521	0.2641
N	0.0023	0.0021	0.0018	0.0014
O	0.3632	0.3728	0.3823	0.4015
Na	0.0234	0.0211	0.0187	0.0140
Mg	0.0000	0.0028	0.0055	0.0111
Al	0.0000	0.0048	0.0097	0.0193
Si	0.0000	0.0176	0.0353	0.0706
S	0.0000	0.0004	0.0007	0.0014
Cl	0.0848	0.0763	0.0678	0.0509
K	0.0149	0.0139	0.0129	0.0109
Ca	0.0000	0.0126	0.0252	0.0504
Mn	0.0000	0.0006	0.0012	0.0023
Ti	0.0000	0.0004	0.0009	0.0017
Cr	0.0000	0.0141	0.0282	0.0564
Fe	0.0017	0.0015	0.0014	0.0010
Co	0.0000	0.0003	0.0006	0.0012
Ni	0.0697	0.0627	0.0557	0.0418
Density (g/cm^3^)	1.134 ± 0.0238	1.217 ± 0.0268	1.326 ± 0.0318	1.56 ± 0.0374

**Table 2:** Compares the linear attenuation coefficient obtained from the experimental results with the values calculated using the MCNP-5 method.

Energy (keV)	Linear Attenuation Coefficient (cm^−1^)											
	E-MW0			E-MW10			E-MW20			E-MW40		
	MCNP-5	EXP	Diff (%)	MCNP-5	EXP	Diff (%)	MCNP-5	EXP	Diff (%)	MCNP-5	EXP	Diff (%)
15	3.600			5.472			7.560			12.811		
20	1.657			2.489			3.419			5.753		
33	0.536			0.740			0.971			1.544		
40	0.391			0.513			0.652			0.993		
59	0.256	0.273	6.492	0.304	0.295	−2.809	0.359	0.368	2.521	0.491	0.511	3.973
70	0.228			0.261			0.301			0.394		
80	0.211	0.193	−8.348	0.236	0.201	−14.917	0.268	0.236	−11.795	0.340	0.286	−15.813
100	0.192			0.211			0.234			0.288		
122	0.178			0.193			0.212			0.255		
244	0.139			0.149			0.162			0.189		
344	0.122			0.131			0.141			0.165		
511	0.104			0.111			0.120			0.140		
662	0.093	0.092	−1.174	0.099	0.094	−5.239	0.107	0.097	−9.649	0.125	0.111	−10.898

**Table 3:** Comparison for the mass attenuation coefficient of the fabricated composites to the mass attenuation coefficient of other similar samples.

Materials	µm (cm^2^/g) at 662 keV
E-MW0 (Present work)	0.082
E-MW10 (Present work)	0.082
E-MW20 (Present work)	0.081
E-MW40 (Present work)	0.08
E@PbO	0.07298
E@CuO	0.06778
E@Halloysite	0.06554
E@Bi_2_O_3_	0.07218
E@Basalt	0.06033
SBR-TiO_2_	0.0258
SBR-Fe_2_O_3_	0.0262
SBR-ZnO	0.0284
SBRMoO	0.0304
Polyster-Bi_2_O_3_ (10%)	0.0798
Polyster-Bi_2_O_3_ (15%)	0.0851
Polyster-Bi_2_O_3_ (20%)	0.0848
HDP	0.079
HDP-nano PbO (50%)	0.114
Polyster-PbI_2_ (10%)	0.082
Polyster-PbI_2_ (20%)	0.0849
Polyacrylamide-ZnO (5%)	0.082
Polyacrylamide-ZnO (10%)	0.081
Polyacrylamide-ZnO (15%)	0.081
Polyacrylamide-ZnO (20%)	0.08
UP-nanoclay	0.074
UP-nanoclay-PbO (10%)	0.078
UP-nanoclay-PbO (20%)	0.083
UP-nanoclay-PbO (30%)	0.084
Per hydro-polysilaxane	0.081
Poly dimethyl silaxane	0.082
Methylsilses quioxane	0.08
Silalkalyene polymer	0.081
Pure Epoxy	0.0832
Epoxy/Al_2_O_3_ (6%)	0.0824
Epoxy/Al_2_O_3_ (15%)	0.0827
Epoxy/Fe_2_O_3_ (6%)	0.0827
Epoxy/Fe_2_O_3_ (15%)	0.0814

## Data Availability

Data are contained within the article.
